# Transglutaminase 6 Is Colocalized and Interacts with Mutant Huntingtin in Huntington Disease Rodent Animal Models

**DOI:** 10.3390/ijms22168914

**Published:** 2021-08-18

**Authors:** Anja Schulze-Krebs, Fabio Canneva, Judith Stemick, Anne-Christine Plank, Julia Harrer, Gillian P. Bates, Daniel Aeschlimann, Joan S. Steffan, Stephan von Hörsten

**Affiliations:** 1 Experimental Therapy, Preclinical Experimental Center, University Hospital Erlangen (UKEr), Friedrich-Alexander-University Erlangen-Nürnberg (FAU), 91054 Erlangen, Germany; fabio.canneva@gmail.com (F.C.); Anne-Christine.Plank@uk-erlangen.de (A.-C.P.); Julia.Harrer@uk-erlangen.de (J.H.); stephan.v.hoersten@fau.de (S.v.H.); 2 Department of Molecular Neurology, University Hospital Erlangen (UKEr), Friedrich-Alexander-University Erlangen-Nürnberg (FAU), 91054 Erlangen, Germany; Judith.Stemick@uk-erlangen.de; 3 Huntington’s Disease Centre, Department of Neurodegenerative Disease and UK Dementia Research Institute at UCL, Queen Square Institute of Neurology, University College London, London WC1N 3BG, UK; gillian.bates@ucl.ac.uk; 4 Matrix Biology and Tissue Repair Research Unit, College of Biomedical and Life Sciences, School of Dentistry, Cardiff University, Cardiff CF14 4XY, UK; AeschlimannDP@cardiff.ac.uk; 5 Institute of Memory Impairments and Neurological Disorders, University of California, Irvine, CA 92697, USA; jssteffa@uci.edu; 6 Department of Psychiatry and Human Behavior, University of California, Irvine, CA 92697, USA

**Keywords:** transglutaminase isoform 6, Huntington disease, neurodegeneration, rodent transgenic animal models

## Abstract

Mammalian transglutaminases (TGs) catalyze calcium-dependent irreversible posttranslational modifications of proteins and their enzymatic activities contribute to the pathogenesis of several human neurodegenerative diseases. Although different transglutaminases are found in many different tissues, the TG6 isoform is mostly expressed in the CNS. The present study was embarked on/undertaken to investigate expression, distribution and activity of transglutaminases in Huntington disease transgenic rodent models, with a focus on analyzing the involvement of TG6 in the age- and genotype-specific pathological features relating to disease progression in HD transgenic mice and a tgHD transgenic rat model using biochemical, histological and functional assays. Our results demonstrate the physical interaction between TG6 and (mutant) huntingtin by co-immunoprecipitation analysis and the contribution of its enzymatic activity for the total aggregate load in SH-SY5Y cells. In addition, we identify that TG6 expression and activity are especially abundant in the olfactory tubercle and piriform cortex, the regions displaying the highest amount of mHTT aggregates in transgenic rodent models of HD. Furthermore, mHTT aggregates were colocalized within TG6-positive cells. These findings point towards a role of TG6 in disease pathogenesis via mHTT aggregate formation.

## 1. Introduction

Huntington disease (HD) is an autosomal dominant disorder caused by the expansion of a CAG repeat stretch located at the N-terminus of the protein huntingtin (HTT) [1]. Expansion to more than 39 CAG repeats causes the typical symptoms described for this disease, characterized by decreased motor coordination leading to involuntary movements, gait instability and rigidity later in life, often preceded by behavioral changes and cognitive deficits [2,3]. Pathophysiologically, mutant huntingtin (mHTT) tends to form cytoplasmic, partly fibrillar aggregates and intranuclear inclusions in humans [4]. So far, the causative sequence of molecular changes leading to neurodegeneration is not fully understood, partly because the contributing pathological mechanisms are not completely elucidated yet. More often, cell death was observed in the presence of monomeric or oligomeric forms of the protein, while neuropil- and soma-associated aggregates seem to be partly protective [5,6,7].

Transglutaminases (TGs) are multifunctional proteins with several enzymatic activities and distinct functions [8,9]. In mammals, at least eight isoforms are known, and for four of these (TG1, TG2, TG3 and TG6) roles in the brain were postulated [9,10,11,12,13]. TG1, the keratinocyte transglutaminase, is mostly known for its essential role in cornified envelope formation in the epidermis [14,15]. TG2 plays a role in a broad spectrum of physiological processes including autophagy, cell adhesion, signal transduction and cell stress response [16,17,18,19] and is a key factor contributing to disease development in patients suffering from celiac disease [20]. The third TG isoform (TG3) is also involved in the formation of the epidermal barrier [21,22] and has a more specialized function affecting epithelial derivatives in a more restricted manner [23]. The precise physiological role of TG6, the neuronal variant, is not known yet. Interactions with polyglutamine proteins have been reported along with hints that it could be involved in neurogenesis and may play an important role in motor-neurons [24] and a mutation in the *TGM6* gene was shown to cause spinocerebellar ataxia 35 (SCA35) [25,26,27]. Recently, TG6 variants were found to differ in patients suffering from Parkinson’s disease, with the wildtype protein having a protective effect on cells by decreasing alpha-synuclein levels and enhancing autophagy [28]. TGs share the common feature of catalyzing irreversible modifications on proteins, including the acyl-transfer between glutamines and lysines [10,16,29]. Under physiological conditions, this transamidation reaction is latent because of low free Ca^2+^ levels. In some pathophysiological conditions, e.g., HD, intracellular free Ca^2+^ may rise [30,31], thus favoring the crosslinking activity of TGs to the expenses of their GTP-binding function, which are mutually exclusive [32].

Several authors have already shown that TG2 activity is increased in patients afflicted with Alzheimer’s disease (AD) and HD as compared to healthy subjects, which suggests that TG2 activity may contribute to the detrimental effects observed in affected individuals [33,34,35]. In support of this hypothesis, Tau protein, amyloid-β peptide (Aβ) and (m)HTT are theoretically excellent substrates for the activity of TGs [34,36,37], which would contribute to enhancing their propensity to aggregation. Nonetheless, the role of TG2 in neurodegenerative disorders is still a controversial subject of discussion [38,39,40,41].

The present study was undertaken to investigate expression, distribution and activity of TGs in the brain of HD transgenic animals [42,43]. We were particularly interested in analyzing the involvement of TG6, as this neuronal isoform is the least characterized in neurodegenerative disorders, yet it is the one abundantly expressed in the central nervous system. The results gathered in HD rodent models indicate a possible contribution of TG6 activity to the pathophysiology of HD.

## 2. Results

### 2.1. Regional Distribution of TG6 in BACHD Mice

The BACHD mouse model was investigated for TG6 expression, as it represents a well-validated animal model of HD pathophysiology in which the full-length human mutant huntingtin protein is expressed [43].

**Figure 1:**
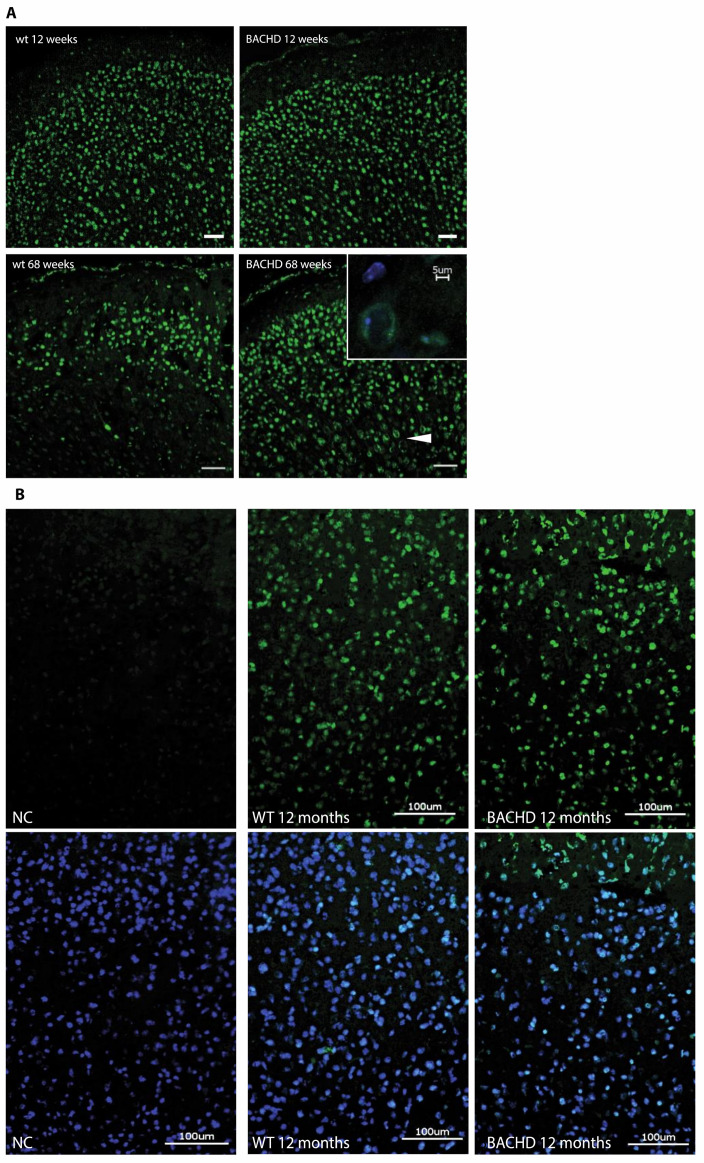
TG6 expression is prominent in the cerebral cortex of BACHD mice. (**A**) Detection of TG6 (green) in the cortices of wildtype (wt) and transgenic (tg) (BACHD) mice by immunofluorescent labeling at 12 and 68 weeks of age. Arrowhead points to distinct TG6 expression pattern in old tg mice. Inlay was taken from the region indicated by the arrowhead. Scale bars: 5, 50 μm. (**B**) In situ assay of TG6 enzymatic activity on cryosections of 12 months old BACHD mice. TG6 activity (green) of wt (center panel) and tg (right panel) animals was determined by incorporation of biotinylated TG6-specific peptide. Nuclei were counterstained using DAPI. Negative controls (NC, left panel) were obtained emitting CaCl_2_ in the incubation buffer. Scale bar: 100 μm.

The distribution of TG6 was prominent and widespread in the brain of 12- and 68-week-old mice, displaying no obvious significant genotype- or age-dependent differences (Figure 1A). In general, the expression of the TG6 protein was highest in the cerebral cortex. Employing immunofluorescence analysis, TG6 distribution appeared to be homogeneously spread throughout the cytoplasm of cortical neurons in wt animals and the soma of larger pyramidal cells located in layers IV and V in the motor appeared more intensively immunolabeled in BACHD tg mice at older age, often displayed a characteristic ring-like (cytoplasmatic) staining pattern often in the periphery of nuclei (Figure 1A, arrowhead, cf. inlay in Figure 1A lower right panel).

### 2.2. TG6 Enzymatic Activity Is Prominent in BACHD Mice

To assess specific TG6 enzymatic activity in situ, we adapted a protocol described by Esposito and colleagues for intestinal tissue [44,45]. As presented in Figure 1B (NC), no or negligible background reactivity was detectable in the absence of CaCl_2_. Conversely, a cellular fluorescent signal (green) was detected very clearly in brain sections of 12-month-old wt and BACHD mice after incubation with the TG6-specific biotinylated peptide in the presence of CaCl_2_, (Figure 1B; center & right panel), although no obvious genotype-dependent differences regarding TG6-specific activity could be detected. Similar results as shown in the cerebral cortex of mice, were also obtained in the olfactory tubercle of old tgHD rats (data not shown). As expected, TG1-, TG2- and TG3-specific reactivity was mostly localized on blood vessels and meninges and no age- or genotype-specific differences were detected in the animal models investigated (Supplementary Figure S1) [45].

### 2.3. Regional Colocalization of TG6 and the TG-Enzymatic Reaction Products in the Brain of tgHD Rats

**Figure 2:**
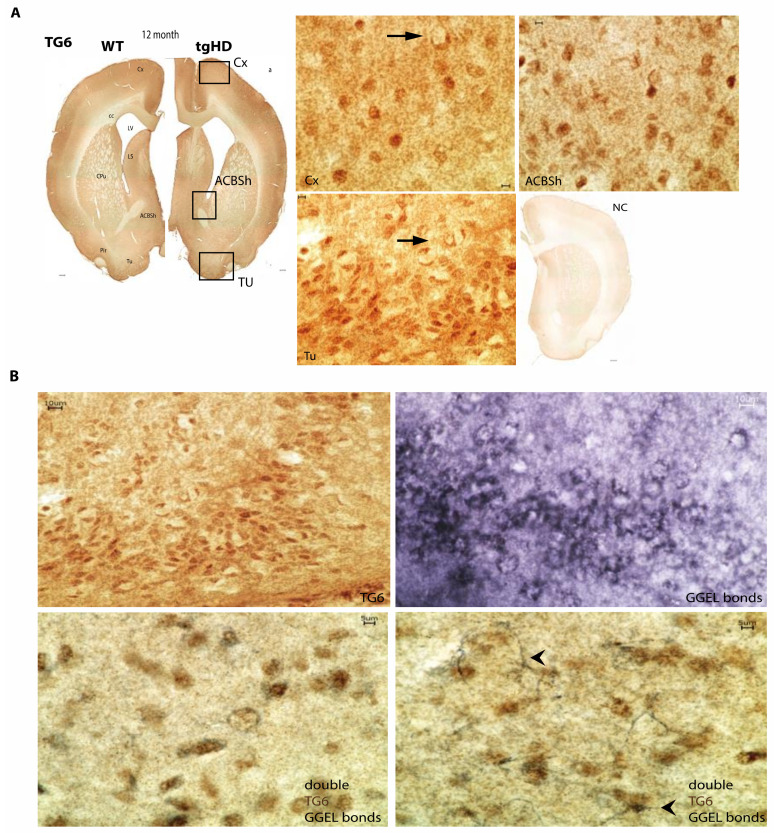
Regional distribution of TG6 and TG-enzymatic reaction products in the brain of tgHD rats. (**A**) The distribution of TG6 is prominent and widespread in the brain of 12-month-old rats, displaying no genotype-dependent differences. The expression level of the TG6 protein was highest in the cortex (Cx; inlay right panel), especially in the piriform cortex (Pir), the lateral septum (LS), the olfactory tubercle (Tu, inlay right panel) and the shell of the nucleus accumbens (ACBSh, inlay right panel). The location of three inlays on the right is indicated within the adjacent tissue overview section (left thumbnail). (**B**) TG6- and 81D4-positive (N-epsilon-gamma glutamyl lysine/GGEL) signals could also be found in neurons. Furthermore, isopeptide bonds could also be found in astrocytes, which appeared in close proximity to TG6 positive neurons (arrowhead). Scale bars: 5, 10, 300 μm.

The regional distribution of TG6 was also investigated in a second rodent model of Huntington disease, using an antibody whose specificity we recently confirmed [45]. Homozygous tgHD rats expressing a truncated 1962 bp Htt fragment with 51 repeats of the polyglutamine stretch under the control of the endogenous Htt rat promoter [42] were analyzed in comparison to wt littermates. As shown in Figure 2A, the distribution of TG6 was prominent and widespread in the brain of 12-month-old rats, displaying no genotype-dependent differences. In comparison, the staining pattern of the ubiquitously expressed TG2 isoform was weaker and the same was observed for TG1 and TG3 (Supplementary data; Figure S1). In fact, compared to these TGs, TG6 was the isoform displaying the most intense signal. Here, the expression of the TG6 protein was highest in the cortex, especially in the piriform cortex, the lateral septum, the olfactory tubercle and the shell of the nucleus accumbens (Figure 2A; right panel). TG6 expression was specifically localized to neurons, but not to astrocytes or microglia, as we previously confirmed using the NeuN-, Iba1- and GFAP-specific antibodies [45]. Furthermore, the reaction products of TGs, the N-ε-(γ-glutamyl)-lysine isopeptide bonds (GGEL) (Dab-Ni, black staining), could also be found in neurons next to astrocytes (Figure 2B), which appeared in close proximity to TG6 (DAB, brown staining) positive neurons (Figure 2B; arrowheads).

### 2.4. Distribution of Mutant Huntingtin Pathology in tgHD Rats

To characterize the expression and distribution of huntingtin and polyQ aggregates in a HD rat and mouse model, brain sections of 16-month old transgenic rats and corresponding wt animal controls were stained either for the presence of non-aggregated endogenous, as well as transgenic, huntingtin (EP867Y) or for intracellular aggregated form (S830) [46]. In comparison to the mice, the transgenic rat HD model is much more suitable for this characterization of mutated huntingtin-dependent aggregation processes, because the pathological lesions are much more abundant and prominent there in comparison to BACHD mice [43,47].

**Figure 3:**
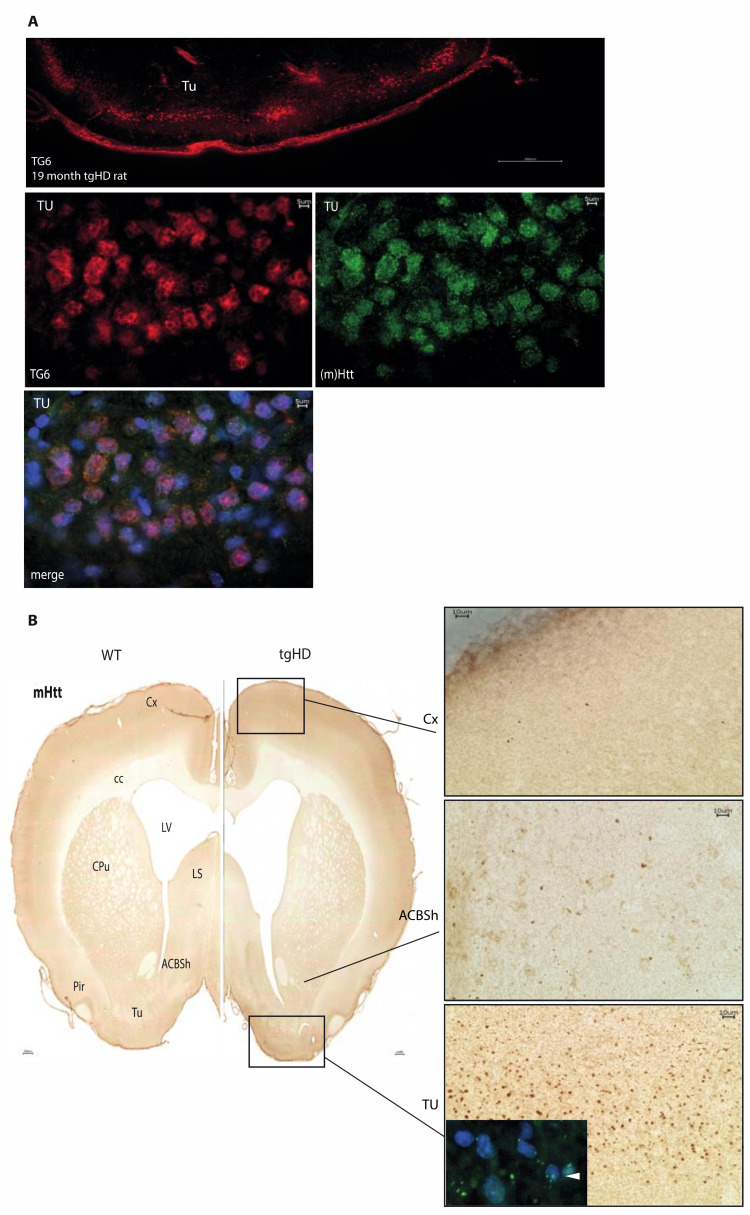
Regional colocalization of TG6 and mHTT aggregates in the brain of tgHD rats (**A**) Immunofluorescent double labeling of TG6 (red) and HTT (green) clearly shows that the two proteins are localized in the same cells, e.g., in the olfactory tubercle (TU). Colocalization was analyzed using a huntingtin- and TG6-specific antibody (EP867Y for HTT, polyclonal goat anti-TG6, both 1:500). Scale bars: 5, 300 μm. (**B**) S830-immunopositive mHTT aggregates display a defined regional distribution pattern. Overview sections of 19-month-old tgHD rats compared to their wt littermates. Areas such as the cortex (Cx), the shell of the nucleus accumbens (ACBSh), the olfactory tubercle (Tu) and the piriform cortex (Pir) are prominent for S830-positive mHTT aggregates (overview section on the left). No staining was found in wt animals. At a higher magnification, S830 immunoreaction products appear as dark or green fluorescent structures, varying in size and form, in the nuclei and cytoplasm (Immunohistochemistry and immunofluorescence images shown in the right panel as indicated by white arrowhead in the inlay). Counterstaining was done using DAPI. Scale bars: 5, 10 and 300 μm.

EP867Y-immunoreactivity confirmed prominent staining of cells in the piriform cortex and the olfactory tubercle, but also in other areas of the brain such as the cerebral cortex and the caudate putamen, without displaying obvious differences between wt and tg rats (Supplementary data; Figure S2A: a, b). Western Blot analysis using the same antibody showed the occurrence of huntingtin fragments of different size in wt and transgenic animals with the truncated transgene running at an apparent molecular size of about 120 kDa (arrowhead in Supplementary data; Figure S2B). Immunoreactivity for TG6 was detected using an isoform-specific antibody. Immunofluorescence double labeling for TG6 and non-aggregated huntingtin clearly showed that the two proteins are localized in the same cells, as detected for example in the olfactory tubercle (Figure 3A).

Immunolabeling of tgHD rat brain sections with the mHTT-specific antibody S830 showed a pronounced and punctuate staining of immunopositive aggregates of varying size and shape, especially in the piriform cortex (Pir) and the olfactory tubercle (Tu) (Figure 3B; overview section on the left). In addition, aggregates could also be found in the cerebral cortex (Cx) (Figure 3B; right panel) and the shell of the nucleus accumbens (ACBSh), typically at lower numbers and smaller in size (Figure 3B; right panel). Aggregated mHTT was undetectable in brain sections of wt littermates (Figure 3B; overview section on the left). In tgHD rats, mHTT aggregates were visible both in the nuclei and in the cytoplasm (arrowhead, Figure 3B; inlay) of cells characterized by a typical neuronal morphology. Furthermore, staining of brain sections from aged transgenic BACHD mice (>15 month) with the S830 antibody confirmed the results obtained by Gray et al. using the EM48 antibody; cortical mHTT aggregates were mostly distributed in the neuropil; however immunoreactivity appeared sparse throughout the cerebral cortex and striatum [43].

### 2.5. Huntingtin Aggregates Colocalize with TG6 in Different Rodent HD Transgenic Animal Models

Based on the evidence of a comparable distribution pattern of the immunoreactivity for TG6, GGELs, mHTT aggregates and huntingtin, we investigated whether TG6 and (m)HTT would directly colocalize and interact by performing ex vivo studies.

**Figure 4:**
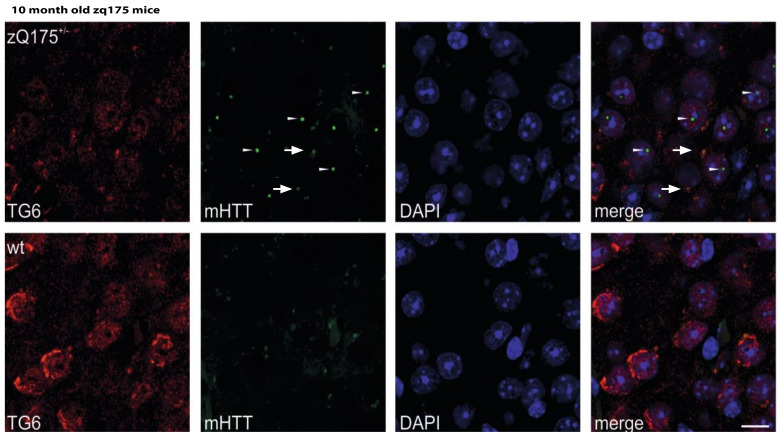
mEM48-immunopositive mHTT aggregates colocalize with TG6. Immunolabeling of brain sections from zQ175DN KI mice with the mEM48 antibody revealed a pronounced and punctuate nuclear staining, particularly in the cortex and the striatum of this HD knock-in mice. Some cells in these areas also showed a colocalized intracellular staining signal with the TG6 isoform (arrows) and mHTT inclusions, as detected with the respective antibodies. Additionally, the TG6 immunolabeling differs between the two genotypes, being more prominent contiguous to the nuclear membrane in wildtype animals. Scale bar: 10 μm.

As already shown, immunolabeling of brain sections from tgHD rats, stained with the S830 antibody, revealed a pronounced and punctuate staining, particularly in the piriform cortex and the olfactory tubercle of HD transgenic rats (Figure 3B). The same results were found using the knock-in zQ175 HD mouse model, which displays higher mutated huntingtin levels and an accelerated phenotype [48]. Using this model, we identified distinctive staining patterns of mEM48 positive mHTT accumulation that showed in the striatum and cortex (Figure 4). Immunolabeling of 10-month-old animals with this antibody showed regional colocalization of TG6 and mutant huntingtin aggregates in cortical areas of these mice (Figure 4, upper panel). mEM48-positive nuclear inclusions, with a distinct punctate staining pattern, were detected in the striatum and cortex (Figure 4, arrowheads). Furthermore, TG6 immunostaining revealed marked differences between the two mouse genotypes, with TG6 labeling more prominent in wildtype mice and more evidently localized to the periphery of the nucleus when compared to knock-in mice. This is consistent with the previously reported localization of TG6 to the nucleus as well as the cytoplasmic side of the ER [49], and suggests that mHTT impacts on TG6 distribution and/or turnover (Figure 4, lower panel).

**Figure 5:**
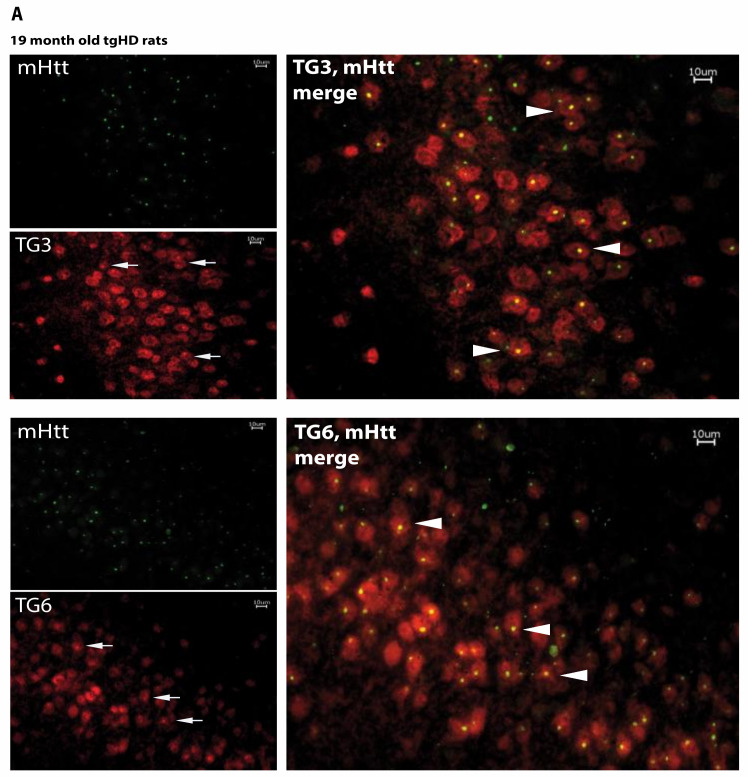

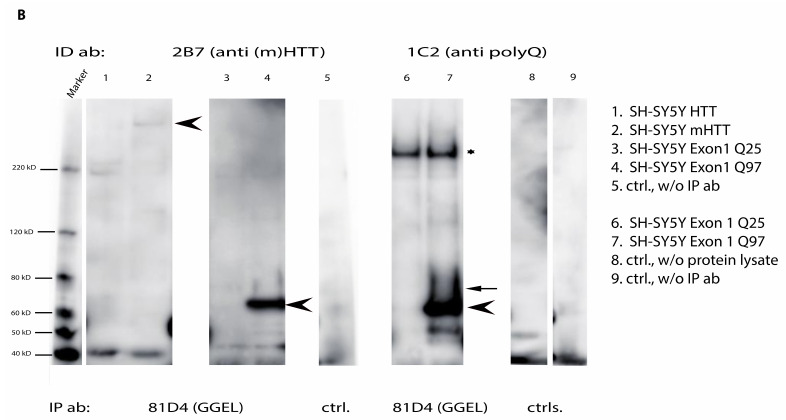
S830-immunopositive mHTT aggregates colocalize with transglutaminase isoforms and are positive for TG-enzymatic reaction products. (**A**) Immunolabeling of brain sections from tgHD rats with the S830 antibody revealed a pronounced and punctuate staining, particularly in the piriform cortex and the olfactory tubercle of HD transgenic rats. Some cells in these areas also showed a colocalized intracellular staining signal with TG isoform 3 and 6 (arrows) and mHTT, as detected with the antibody S830 (arrowheads). Scale bars: 10 μm. (**B**) mHTT protein and exon 1 Q97 fragment are positive for N-ε-(γ-L-glutamyl)-L-lysine (GGE L) bonds. Immunoprecipitation of GGEL-modified (m)HTT proteins and exon 1 fragments by the 81D4-antibody in total protein lysates of transfected SH-SY5Y cells. Blots displayed specific differences regarding the presence of GGEL-modified mutated full-length huntingtin or exon 1 Q97 as detected by the HTT-specific antibody 2B7 (CHDI) (1:2000) or an antibody directed against a prolonged polyglutamine stretch (1C2, MerckMillipore Darmstadt) (1:1000). Arrow and arrowheads indicate either full-length mutated huntingtin (about 350 kDa) or exon 1 Q97 (about 70 kDa), asterisk indicates unspecific signal using Protein L Agarose (Santa Cruz Biotechnology, Heidelberg, Germany) for immunoprecipitation.

This result was confirmed in tgHD rats, where TG6 expression was also most prominent in these areas. Fluorescent double labeling with the S830 antibody and TG3- or TG6-specific antibodies clearly revealed colocalization of the proteins, with most mHTT aggregates being double-positive for S830 and TG6 immuno-reactivity (Figure 5A, arrows). Noteworthy, TG3 staining was also observed in the same brain areas and displayed a similar colocalization behavior with the S830-positive lesions (Figure 5A, arrowheads). No colocalization of the mHTT aggregates with TG1 or TG2 was observed (data not shown).

### 2.6. Mutant Huntingtin Contains Transglutaminase-Catalyzed Crosslinks

The occurrence of GGELs in full-length (m)HTT or exon1 fragments was investigated in protein lysates of transiently transfected cells after immunoprecipitation with the mAb 81D4. At first and to prove expression of both exon 1 variants in transiently transfected SH-SY5Y cells, we performed Western blot and immunoprecipitation analysis using an antibody directed against the EGFP tag followed by detection with a huntingtin-specific antibody. Therefore, we were able to prove expression of the exon 1 fragments (Figure S3).

We could show differences regarding the presence of GGEL-altered full-length huntingtin or the exon 1 fragments in transfected SH-SY5Y cells (Figure 5B). As shown in Figure 5B, isopeptide crosslinks were found in full-length mutated huntingtin as well as in an exon 1 fragment bearing a polyglutamine stretch with *n* = 97 glutamines, after immunoprecipitating isopeptide-modified huntingtin using the 81D4 antibody. This was true for both the detection with an antibody directed against the N-terminus of the huntingtin protein (2B7), therefore recognizing both the wildtype and mutated HTT variant, or an antibody detecting only the prolonged polyglutamine stretch (1C2) (Figure 5B, arrowheads). Furthermore, some faint bands, possibly representing smaller fragments of the full-length huntingtin proteins were also visible, regardless of the length of the polyQ stretch. Additionally, immunolabeling of the exon 1 fragment harboring 97Qs displayed a smeary pattern (Figure 5B, arrow), possibly indicating an isopeptide-modified exon 1 fragment.

Isopeptide covalent crosslinks were also found in full-length rat huntingtin. For the full-length rat huntingtin protein, this modification was irrespective of genotype and age of the animals (data not shown).

### 2.7. Direct Interaction of TG6 and Huntingtin

To elucidate if TG6 is directly interacting with HTT, and possibly responsible for its posttranslational modification, co-immunoprecipitation studies were performed. Protein lysates of transiently double transfected SH-SY5Y cells and brain protein lysates of 55 weeks-old wt or transgenic rats, were analyzed for interaction of full-length (m)HTT and exon 1 fragments with TG6. For the detection of TG6, either a TG6-isoform-specific antibody [45] or an antibody detecting the STREP II-tag connected to the transfected TG6 protein was used.

**Figure 6:**
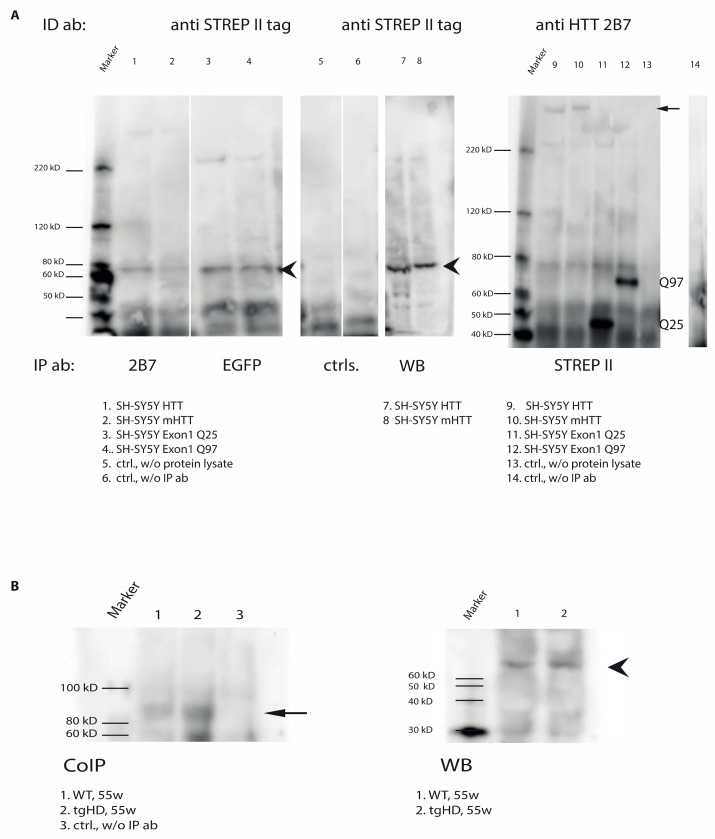
Direct interaction of TG6 and (m)huntingtin. (**A**) For interaction analysis of (m)HTT and exon1 fragments with TG6, protein fractions of transiently transfected SH-SY5Y cells, expressing either full-length (m)HTT or exon1 fragments with either a normal (25 polyglutamines) or mutated (97 polyglutamines) CAG repeat connected to a C-terminal EGFP were used. Anti-Strep II antibody was used for the identification of TG6. (**B**) TG6 was co-immunoprecipitated from the soluble fractions of both wt and tgHD rats (arrow). Brain protein lysates of wild type and tgHD rats (55 weeks-old) were used and (m)HTT was precipitated using a (m)HTT-specific antibody (mab2166, MerckMillipore, Darmstadt, Germany; 1 μg). For the detection of TG6, a polyclonal isoform-specific antibody (Zedira, Darmstadt, Germany) was used. The Clean-Blot IP Detection reagent (Thermo Scientific, Waltham, MA; USA), coupled to HRP, was used to reduce interference from denatured IgG background signal. The TG6 protein displayed the expected molecular weight of about 79 kDa when analyzed by Western blot (WB, arrowheads).

TG6 was co-immunoprecipitated from the soluble fractions of SH-SY5Y cells expressing either the full-length huntingtin variants or the exon 1 fragments, regardless of the length of the polyglutamine stretches (Figure 6A, left panel, arrowheads). Vice versa, by performing a co-immunoprecipitation using the STREP II-tag antibody to pull-down TG6, full-length huntingtin next to exon 1 fragment was co-precipitated, again irrespective of the length of the polyglutamine stretch (Figure 6A, right panel, arrow). Furthermore, TG6 was co-immunoprecipitated from brain lysates of wt and mHTT transgenic rats (Figure 6B, CoIP, arrow), again suggesting a direct interaction between (m)HTT and TG6 also in vivo. The TG6 protein displayed the expected molecular weight of about 79 kDa when analyzed by Western blot, thus reflecting the specificity of the antibody used (Figure 6B, WB, arrowhead).

### 2.8. TG6 Overexpression Induces Accumulation of Mutant Huntingtin Exon 1 Aggregates

**Figure 7:**
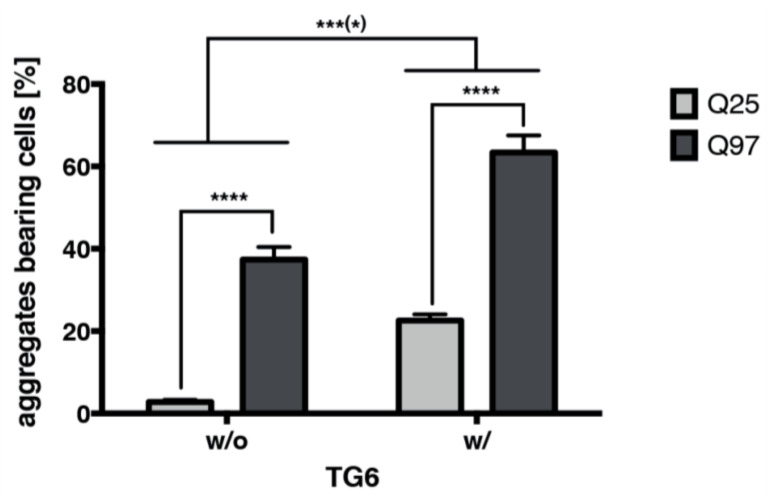
TG6 enzymatic activity increases the total amount of mutant huntingtin exon 1 aggregates. For quantification of mutant exon 1 GFP aggregates, SH-SY5Y cells were transiently (double) transfected with the exon 1 variants and with (w/) or without (w/o) an enzymatically active TG6 protein. Percentage of transfected cells containing aggregates for each of the exon 1 GFP fragments was quantified. Data are mean ± SEM of three independent experiments counting at least 200 cells per approach. Approaches were done in triplicates. For statistical analysis two-way ANOVA and Bonferroni post-test were used. Statistical significance is indicated by *** *p* < 0.001, **** *p* < 0.0001., respectively.

Consistent with the direct interaction of TG6 and (m)HTT, we found that TG6 overexpression in double transfected SH-SY5Y cells accelerates the amount of huntingtin exon 1 GFP aggregates (Figure 7). Representative images showing double transfected aggregate-bearing SH-SY5Y are provided in Supplementary Figure S4. Furthermore, in SH-SY5Y cells TG6 not only increases the quantity of insoluble aggregates in comparison to cells expressing only the mutant exon 1 fragment, the total amount of mutant exon 1 aggregates was significantly raised by TG6 enzymatic activity. Notably, only a few inclusions were visible in cells expressing an exon 1 fragment without a prolonged polyglutamine stretch.

## 3. Discussion

The present study demonstrates the physical interaction between TG6 and huntingtin in vitro and describes TG6 expression and transamidation activity ex vivo in rodent HD animal models in those brain areas where neurons display highest amounts of mHTT aggregates. These findings represent an original observation strongly supportive for a role of TG6 in the formation of mHTT aggregates.

Up to now, transgenic mouse models of HD are by far the most studied models for HD and are widely used and accepted [50,51]. Although long and cost-intensive, research into disease mechanisms is an important basis for the development of novel, targeted therapies in translational research. Therefore, suitable animal models which allow predictions on the efficacy and safety of novel therapies are inevitable in this process. A successfully established animal model of a neurodegenerative disease adequately recapitulates the human disorder, both mimicking the symptomatology and developing adequate neuropathological lesions. Recently a transgenic minipig model for HD was established and characterized [52,53,54]. Although pig models are believed to be superior to mouse models with respect to recapitulation of human disease phenotypes, and technologies for generating genetically modified pig models have been well established, there are several reasons that hamper the broad applications of pigs [55].

In our study, the distribution pattern of mHTT aggregates was confirmed in tgHD rats using the S830 antibody, a well-characterized antibody reactive to fibrillar, aggregated mHTT material [56]. Immunolabeling of brain sections with this antibody showed a pronounced and punctuate staining of mHTT-immunopositive-aggregates, especially in the piriform cortex and the olfactory tubercle of HD transgenic rats. The knock-in zQ175 mice and the transgenic HD animal models BACHD mice and tgHD rats [42,43,48,57] are characterized by a progressive, age-dependent development of behavioral as well as histochemical hallmarks typical for HD disorder. However, the zQ175 mice and the tgHD rats were more suitable for the validation of our findings than the BACHD mice, because the pathological lesions are more abundant and prominent in these models [42,47,48,57,58]. They allow the investigation of molecular events, specifically targeting pre- and early-symptomatic stages of disease progression in comparison to a later fully developed HD-like phenotype, including the prominent accumulation of mHTT aggregates (reviewed in [50]). In particular, the tgHD rat is characterized by an early anxiolytic-like phenotype, already detectable at the age of 8 weeks, followed by progressive cognitive decline along with motor impairment, which are obvious by the age of 40 weeks. Additionally, premature psychomotor responses [59] as well as involuntary (chorea-like) movements are visible in rats at about 60 weeks of age [60]. However, only rats of advanced age (>18 month) are known to exhibit prominent deposition of mHTT aggregates, whereas rats younger than 18 months have been reported to show milder neuropathological symptoms and only sporadic aggregates [42,47]. Furthermore, using the EM48 antibody, Petrasch-Parwez et al. found mHTT aggregates of varying size and forms, which were identified in neuronal nuclei, the cytoplasm, dendrites, dendritic spines and synaptic terminals. Brain samples from 3–12 months old zQ175 heterozygous mice were stained for mHTT inclusions also using the mEM48 antibody by another group [57]. An age-dependent progressive increase of the mEM48 signal, in both striatal and cortical brain regions, was clearly visible with mHTT inclusions appearing earlier and with higher abundance in the striatum as compared to cortex in the zQ175 mice [57].

We chose the zQ175DN KI and not the R6/2 model, because they express the full-length protein in which the mouse Htt exon 1 has been replaced by the human HTT exon 1 sequence with a ~190 CAG repeat tract with spread nuclear and neuropil inclusions [57,61]. In contrast to zQ175 DN KI mice, the R6/2 mice model human HD by expressing only a portion of the human HD gene under human gene promoter elements. The expression of this N-terminal exon 1-fragment of the huntingtin protein with its polyglutamine expansion is sufficient to produce a full HD-phenotype in a very short time. Therefore, knock-in mice are very useful for studying Huntington disease pathogenesis of the juvenile HD-phenotype without hardly showing any survival rate limitation, especially in heterozygous animals.

In contrast to the afore mentioned models, BACHD transgenic mice display a significant and progressive motor impairment starting at 8 weeks of age and mHTT inclusions are predominantly found in neuropil (48w) but only a few are visible in the cortex and striatum of this model (72w) [43]. Furthermore, aggregate-bearing cells are sparse in heterozygous mice thus making them less suitably for studies of aggregation-associated processes. We believed that heterozygous knock-in models best mimic the human condition from a genetic perspective since they express the mutation in the appropriate genetic and protein context and published data support the suitability of this model for the evaluation of pathogenic events during HD [61].

The aim of our study was to widen our understanding on the role of TGs in the pathogenesis of HD, as discussed for other neurodegenerative disorders [38,39,40,41,62]. In vitro experiments by Zainelli and colleagues suggested the involvement of the TG isoforms 1, 2 and 3 in HD [36]. The topic is especially interesting and sensitive since TG-mediated reactions are essential for many biological processes and are involved or suggested to be involved in several human diseases, such as skin barrier formation failure in lamellar ichthyosis [21,63], peptide modification driving autoimmunity in celiac disease [64,65], blood coagulation disturbances [66] and neurodegenerative disorders [33,67,68,69], where TG transamidating activity is accompanied by an increase of TG-catalyzed products [70,71]. Recently, TG6 turned into the focus of many researchers by showing its association with neurogenesis and neuronal differentiation [24]. Additionally, in spinocerebellar ataxia 35 (SCA35), a mutation in the *TG6* gene was shown to be causative of the disease [25,26]. Furthermore, it was demonstrated that the direct interaction of TG6 with polyglutamine proteins could promote the formation of polyglutamine aggregates [27].

Here, we could demonstrate that all known four TG isoforms found in the brain are expressed in brains of tgHD rats. Prominent fluorescence signals of all four proteins were found in vessels, the meninges and the ependyma. The most prominent staining signals for TG6 were detected in neurons of the lateral septum, the cortex, the piriform cortex and the olfactory tubercle and the last two regions exhibited the strongest staining signals for TG1, TG3 and TG6. Our results are comparable to the regional TG6 distribution found by others in C57BL/6J mice [72]. Nevertheless, the expression of the TG6 protein was most prominent in those regions where HTT is also highly expressed, both in wt and tg rodents. Indeed, the piriform cortex and the olfactory tubercle are the regions that exhibited the highest concentrations of mHTT aggregates next to non-aggregated huntingtin in HD transgenic animals of advanced age. As already mentioned, in our rat animal model the S830 antibody nicely stained mHTT lesions but only TG3 and TG6 appear to colocalize with aggregated mHTT, demonstrating physical proximity of the proteins. Noteworthy, not every mHTT aggregate colocalized with the TG3- or TG6-signal, but where colocalization occurred, the aggregates displayed a round shape with a nuclear location. These double-positive structures were most prominent in the piriform cortex and the olfactory tubercle but could also be found in other areas of the cerebral cortex and the nucleus accumbens shell of tgHD rats. In all cases, mHTT aggregates were present where prominent expression of the TGs, especially TG6, was found. Comparable results were found in a knock-in mouse model of HD. However, these animals displayed marked differences regarding TG6 distribution, with wt littermates showing a prominent TG6 immunolabel contiguous to the nuclear envelope, probably the endoplasmic reticulum. The endoplasmic reticulum is one origin of the autophagosome membrane in mammals [73] and a distorted autophagy process is a hallmark of neurodegenerative disorders such as Parkinson’s, Alzheimer’s and Huntington disease [74,75,76]. Autophagy is a highly conserved cellular process responsible for the degradation of misfolded proteins. Recently, the wildtype variant of transglutaminase 6 was found to protect cells by decreasing alpha-synuclein and enhancing autophagy during Parkinson’s disease [28], pointing towards a prominent role of TG6 for the autophagy process, possibly for autophagosome maturation, which is lost during HD pathophysiology. A role for the TG2 isoform, the most prominent member of the transglutaminase protein family, for the maturation of autophagosomes was already shown [18,19].

The localization of TG6-specific enzymatic activity (as visualized by our in situ activity assay) further confirmed our results, showing a similar distribution as obtained by IHC, with a clear abundance in the soma of cells located in the cerebral cortex and the olfactory tubercle. In agreement with the in situ detected TG6-based enzymatic activity is the fact that enzymatically active TG6 can increase the aggregate load in double transfected SH-SY5Y cells, in comparison to cells expressing the mutated huntingtin exon 1 fragment alone, thus further pointing towards a key role of TG6 for the pathophysiology of HD.

Transglutaminases catalyze the irreversible posttranslational modification of proteins [77,78]. The transamidating activity of TGs is activated through the binding of Ca^2+^. Calcium dyshomeostasis is a common event during pathophysiological processes of neurodegenerative diseases [79,80,81], and huntingtin, with its expanded polyglutamine stretch, is an ideal candidate substrate for transglutaminases. Indeed, GGEL-immunopositive cells were abundant in the piriform cortex and olfactory tubercle in HD transgenic animals. Noteworthy, GGEL-reactivity was also detected in astrocyte-like cells, although TG6 was not found to be expressed in astrocytes or microglia of adult mice and in our rats [45,72], indicating that those structures may be the reaction product of another TG isoform, probably TG2, which is found in these cell types [82]. Here, we have demonstrated the presence of GGEL-modified mutated full-length huntingtin and exon 1 fragment in total protein lysates of transfected SH-SY5Y cells. Furthermore, in vivo full-length huntingtin also appeared to contain GGEL modifications, albeit irrespective of the age and genotype in transgenic rats. In this context, it seems obvious that TG6 binds (m)HTT from transfected cell lines as well as in WT and tgHD rats at yet not defined levels (Figure 6A,B). As coIP is not quantitative and since full-length as well as N-terminal fragments of HTT as well as mHTT were immuno-precipitated by TG6, we can conclude at this point that TG6 binds N-terminal HTT and mHTT regardless of the length of the polyQ stretch. Thus, it should be kept in mind that (a) based on the direct evidence provided here, the length of the polyQ stretch does not play in role in TG6 interaction (although the (m)HTT-ID-antibody mAB2166 has—according to manufacturer information—a higher affinity for HTT), that (b) purified (m)HTT fragments are not available for qualitative/quantitative interaction studies at present, that (c) through using different antibodies (2166, S830, 2B7, EGFP) (cf. supplementary Table S1) all evidence accumulates that the site of interaction is found in exon 1, and that (d) all-in-all, it may well be that the affinity for mHTT by TG6 is higher though further studies are required. In general, the signal intensity was faint and an exon 1 fragment in transgenic animals was not detectable. However, although we could not detect genotype-dependent differences in TG6-dependent enzymatic activity in vivo, it is still possible that protein modifications due to low rates of TG6-specific enzymatic activity are sufficient to contribute to HD pathophysiology. Such soluble aggregates have been postulated to drive the disease process [83].

The fragmentation of full-length (mutated) huntingtin into peptides of lower molecular weight has been described in detail and demonstrated in an HdhQ150 knock-in mouse model of HD as well as in their wt littermates, although the number of fragments is increased in the transgenic mice [84,85]. In that study, HTT fragments were detected in animals of all ages and did not appear to represent stages of the pathogenic progression. Furthermore, Landles et al. demonstrated that the smallest fragment produced originated from the sequence coded on exon 1 of the *HTT* gene and that such peptides, possibly along with other fragments, specifically accumulated in neuronal nuclei, prior to the onset of any behavioral phenotypes [84]. Furthermore, the subcellular localization and formation of huntingtin aggregates correlates with symptom onset and progression in a R6/2 mouse model with 90 CAGs [85]. As with its fragmentation, GGEL-bonds formation on HTT and possibly smaller N-terminal fragments, may be a physiological modification, which acquires a pathological value when occurring on fragments derived from genetically altered portions of the molecule. Since the immunoprecipitation of mHTT with a GGEL-specific antibody does not necessarily demonstrate the involvement of TG6 and its direct interaction, we decided to perform co-immunoprecipitation experiments in which TG6 or the HTT proteins were co-immunoprecipitated from protein lysates obtained from wild type and transgenic rats or transfected SH-SY5Y cells. The results obtained suggested the direct interaction of TG6 and (m)HTT. However, we cannot rule out the possibility of the existence of a larger complex that also contains these two proteins, because TGs do not only interact at the crosslinking site with their substrates but the interaction could also be guided by independent bindings site(s) [86]. Specificity of the used TG6 antibody was recently confirmed by us using purified TG proteins [45]. Thus, our results point towards an involvement of transglutaminases, especially TG6, in the formation of soluble, oligomerized (mutated) huntingtin oligomers. This hypothesis is in good agreement with the results presented by others, where neuronal TG6 was shown to promote the formation of polyglutamine aggregates by the conversion of soluble polyglutamine proteins into insoluble aggregates [27].

## 4. Materials and Methods

### 4.1. Animal Models

Homozygous tgHD rats and littermate wild type controls [42], expressing a 1962 bp *HTT* fragment with 51 repeats of the CAG stretch (mHTT) under the control of the endogenous *HTT* rat promoter, were used in this study. Transgenic BACHD mice expressing full-length human mutant huntingtin (mHTT) with 97 polyglutamine repeats [43] were used as a second species HD model. Male wild type (wt) and transgenic (tg) mice and rats were characterized at different age points representing different disease stages with at least three individuals per genotype and age. Male heterozygous zQ175DN KI knock-in mice with 180–220 CAG repeats and wildtype littermate controls were acquired from Jackson Lab for histology studies. Colonies of rats and mice were established at the preclinical research center of the Universitätsklinikum Erlangen. All animals were kept under a 12:12 h light: dark cycle (lights on at 6.00, off at 18.00) with food (Ssniff lab chow pellets; Soest, Germany) and tap water available ad libitum. Rats and mice were housed in gender- and genotype-matched groups of four according to FELASA recommendations. All research and animal care procedures were approved by the local district governments (#54-2532.1-19/09, TS-1/08) and performed according to international guidelines for use of laboratory animals. For genotyping, tail biopsies were collected at the age of 3 weeks.

For brain collection, animals (5 wt vs. 5 tg) were deeply anesthetized with a mixture of Ketamine and Xylazine [87] and quickly perfused with ice-cold saline solution (0.2 M Phosphate buffer, supplemented with 137 mM NaCl, 3 mM KCl and 6 mM NaHCO_3_). Post-perfusion, brains were fixed overnight in 4% paraformaldehyde solution (PFA; pH 7.5), transferred to 30% sucrose in phosphate buffered saline (PBS; Biochrom AG, Berlin, Germany) for min. 48 h and finally snap-frozen in iso-pentane for 30 s at −60 °C and stored at −80 °C until use. Tissue assigned for the investigation of TG activity was frozen immediately (without fixation) in iso-pentane (−60 °C) and stored at −80 °C.

### 4.2. Plasmid Constructs

Expression vectors for full-length human huntingtin were synthesized at GenScript. Full-length wildtype (wt) HTT contains 23 consecutive CAG repeats. Mutant huntingtin (mHTT) with 90 polyglutamines, was cloned by inserting further CAG repeats into the wildtype cDNA. Afterwards, both cDNAs were cloned into expression vector pcDNA3.1. The constructs encoding the exon 1 fragment of HTT, containing 25 or 97 Qs, were previously cloned into pcDNA 3.1 [88].

The TG6 expression plasmid is under the control of a constitutive promoter (CMV). The plasmid is a modified version of pCEP4 and contains TG6 with a C-terminal Strep2 tag, located between AflII and NotI [24].

### 4.3. Cell Lines and Cell Culture

SH-SY5Y cells (ATCC) were maintained in DMEM medium supplemented with 10% FBS in a humidified incubator at 37 °C with 5% CO_2_. We used Lipofectamine 2000 (Invitrogen, Carlsbad, CA, USA) to transiently transfect cells, according to the manufacturer’s instruction.

### 4.4. Quantification of Mutant Huntingtin Exon 1 Aggregates and Statistical Analysis

Mutant GFP-exon 1-based aggregation was monitored with the Keyence fluorescence microscope. A total of 24 h after transfection cells very fixed using 4% PFA. When using GFP-exon 1 variants either with or without enzymatically active TG6, at least 200 (double) transfected cells were counted per coverslip and the proportion of cells with at least one aggregate/inclusion was scored as a percentage of the total number of transfected cells. The experiments were performed without knowing the identity of the slides at three times in triplicates.

Presented data are mean ± SEM of three independent experiments counting at least 200 cells per coverslip using the Prism6 software. For statistical analysis two-way ANOVA and Bonferroni post-test were used.

#### 4.4.1. Glutamine-Donor Synthetic Peptides

Transglutaminase-isozyme-specific glutamine-donor substrate peptides were identified and tested for their isozyme-specificity by Kiyotaka Hitomi et al. [89,90,91,92]. The respective sequence for TG6 is Biotinyl-Aca-DDWDAMDEQIWF and was synthesized by Genaxxon bioscience GmbH (Ulm, Germany). Here, the biotin was separated from the peptide by epsilon-aminocaprionic acid (Aca), a chemical inert linker, which enhances the availability of the biotin for streptavidin.

#### 4.4.2. In Situ TG Activity Assay on Brain Section

To establish an optimized in situ brain TG isoform-specific enzymatic activity assay on brain sections, we adapted a protocol described by Esposito et al. in 2003 for use on duodenal bioptic tissue [44,45].

Briefly, frozen, unfixed brains were sliced into 10 μm-thick coronal sections on a cryostat (CM3050S, Leica Biosystems, Nußloch, Germany) and mounted on glass slides. After air-drying for 60 min at room temperature, sections were gently fixed in −20 °C cold acetone for 10 min, followed by incubation with 1% bovine serum albumin (BSA) in 0.1 M Tris/HCl (pH 7.4) for 30 min, rinsed once in PBS and then treated with Avidin-Biotin-Block solutions (Dako, Hamburg, Germany) following the manufacturer’s instructions. Sections were then incubated for 2 h in the presence of 0.1 mmol/L biotinylated TG isoform-specific peptide in 0.1 M Tris/HCl, 5 mM CaCl_2_ (pH7.4). Control sections were incubated in the absence of CaCl_2_ in the incubation buffer or without biotinylated peptide. Reactions were stopped with 25 mM EDTA in PBS for 5 min. Slides were then washed three times with PBS and incorporation of biotinylated peptides visualized by incubation with streptavidin avidin fluorescein isothiocyanate (1:50; Dako) for 30 min at RT. Nuclei were visualized using DAPI and background autofluorescence (as caused by lipofuscin bodies in specimens from older animals) was darkened by treatment with 0.1% Sudan Black (15 min, RT). Finally, brain sections were air-dried for 1 h, and mounted with Vectashield Mounting medium.

### 4.5. Immunohistochemistry and Immunofluorescence

Frozen, fixed brains were sliced into 40 μm-thick coronal sections on a cryostat (CM3050S, Leica Biosystems, Nußloch, Germany). The sections were then stained using a free-floating immunohistochemistry procedure. PBS-T (PBS containing 0.2% TritonX-100) was used throughout all passages and incubations were all performed at room temperature, unless otherwise specified. Briefly, tissue was permeabilized with PBS-T and then treated with 0.3% hydrogen peroxide for 20 min, rinsed and blocked for 60 min with 5% normal donkey serum (Jackson ImmunoResearch Europe, Ely, Cambridgeshire, UK). Incubation with primary antibodies (monoclonal rabbit anti-huntingtin EP867Y, 1:500 (Epitomics, Cambridge, UK); sheep-anti-huntingtin S830, 1:10,000; polyclonal rabbit anti-TG6, 1:500 (Zedira, Darmstadt, Germany); polyclonal anti-GFAP, 1:20,000 (Dako, Hamburg, Germany); monoclonal anti-NeuN, 1:50,000 (MerckMillipore, Darmstadt, Germany); mouse monoclonal 81D4 to N-epsilon-gamma glutamyl lysine (GGEL), 1:250 (Abcam, Cambridge, UK)), diluted in REAL antibody diluent^®^ (Dako, Hamburg, Germany) was performed overnight at 4 °C. The following day sections were rinsed and incubated for 1 h with the secondary antibody (donkey-anti-rabbit IgG, 1:500; donkey-anti-mouse IgG, 1:500; Santa Cruz Biotechnology, Heidelberg, Germany, donkey-anti-mouse IgM, 1:500, Jackson ImmunoResearch, Ely, Cambridgeshire, UK). Finally, sections were washed and incubated for 30 min with VECSTASTAIN^®^ ABC reagent (Vector Laboratories, Peterborough, UK). The staining was developed with Ni-enhanced DAB reagent or DAB (Vector Laboratories, Peterborough, UK). After extensive washing, sections were blocked with streptavidin- and biotin-blocking agents (Dako, Hamburg, Germany).

For double labeling, sections were incubated overnight with the second primary antibody (mouse monoclonal 81D4 to N-ε-(γ-L-glutamyl)-L-lysine (Abcam, Cambridge, UK), 1:500). Next day, sections were rinsed and incubated for 1 h with the corresponding secondary antibodies (biotinylated donkey-anti-rabbit IgG, biotinylated donkey-anti-sheep or biotinylated donkey-anti-mouse, 1:500; Santa Cruz Biotechnology, Heidelberg, Germany or Jackson ImmunoResearch, Ely, Cambridgeshire, UK). The staining was developed with DAB reagent or SD-reagent (Vector Laboratories, Peterborough, UK). Rinsed sections were then mounted on Superfrost Plus glass slides (Thermo Scientific, Waltham, MA, USA), air-dried overnight, dehydrated in ascending ethanol concentrations, cleared with xylol and coverslipped with DPX mounting medium (Sigma, Munich, Germany). All images were acquired on a Keyence BZ9000E microscope, equipped with imaging software (Keyence, Neu-Isenburg, Germany).

For immunofluorescence, tissue was prepared and treated mainly according to the protocol described above. The following primary antibodies were used: monoclonal mEM48 anti-huntingtin ab (MAB5374, MerckMillipore, Darmstadt, Germany), 1:100; monoclonal rabbit-anti-huntingtin EP867Y, 1:500 (Epitomics, Cambridge, UK); polyclonal anti-TG3 or -TG6, 1:1000 or 1:200 for IF studies on zQ175 mice tissue (Zedira, Darmstadt, Germany); sheep-anti-huntingtin S830, 1:1000) and incubated overnight at 4 °C. A summary of the primary antibodies is provided in Supplementary Table S1. To enhance the TG-fluorescence signals of the antibodies, the TSA Plus Biotin Kit (Perkin Elmer, Waltham, MA, USA) was used according to the manufacturer’s protocol. After rinsing in TBST, appropriate secondary antibodies (Alexa 488, Alexa 568, and Alexa 647) were diluted in blocking solution and applied for one hour at RT, followed by three to five more washing steps and nuclear staining using DAPI solution (1:1000, Sigma Aldrich, St. Louis, MO, USA). High resolution Z-stack fluorescence images were acquired as 1 μm steps on a Keyence BZ9000E microscope or Zeiss Confocal microscope with 60×- and 100× objectives, equipped with imaging software.

Since lipofuscin autofluorescence was prominent in brain sections of older animals, sections received an additional treatment with 0.1% Sudan Black B in 70% Ethanol (Merck, Darmstadt, Germany) for 15 min at RT. After final washings, sections were mounted, air-dried and coverslipped in Vectashield Mounting medium.

### 4.6. Sample Preparation of Brain Tissues or Transfected Cells for Immunoblotting

Brain specimens collected from experimental animals were stored at −80 °C. Midbrain samples or transfected cells were homogenized at 4 °C in IP Lysis-buffer (50 mM Tris-HCl, 150 mM NaCl with 1% Tween-20 (Sigma Aldrich, St. Louis, MO, USA), pH 7.5) containing protease inhibitors (Complete protease inhibitor cocktail, Sigma Aldrich, St. Louis, MO, USA).

All preparations were centrifuged at 10,000× *g* for 30 min at 4 °C to remove cell debris. Supernatants were collected and protein concentrations determined by means of the Bradford method according to the manufacturer’s protocol (Roti-Quant, Carl Roth GmbH, Karlsruge, Germany).

### 4.7. Co-Immunoprecipitation

Immunoaffinity purification of proteins containing GGELs was performed using Protein L Agarose beads (Santa Cruz Biotechnology, Heidelberg, Germany) for the 81D4 mAb. Briefly, 50 μL of Sepharose beads were mixed with 800–1000 μg of whole protein lysate of transiently transfected cells and incubated at 4 °C for 2 h. After centrifugation at 2000× *g* for 2 min at 4 °C, the supernatant was discarded and the pellet was washed 4 times in 1.0 mL TBS (20 mM Tris, 140 mM NaCl, pH 7.5) with 0.1% Tween 20 (TBST). Each bead-pellet was then resuspended in 25 μL of 1× LDS-sample loading buffer (Invitrogen, Carlsbad, CA, USA) and boiled at 90 °C for 5 min. The antigens were separated from the Agarose beads by centrifugation at 2000× *g* for 2 min at RT. GGEL-modified huntingtin fragments were identified using the huntingtin-specific 2B7 antibody [93] (1:2000) or an antibody recognizing a prolonged polyglutamine stretch (1C2; 1:1000; MerckMillipore, Darmstadt, Germany).

For co-immunoprecipitation of (m)HTT and HTT exon 1 fragments with TG6, transiently transfected SH-SY5Y cells, expressing either full-length (m)HTT or an exon 1 fragment with either a normal (polyglutamine with *n* = 25 glutamines) or mutated (polyglutamine with *n* = 97 glutamines) CAG repeat connected to a C-terminal EGFP, as well as half-brains of 55 weeks-old wild type (WT) and transgenic animals (tgHD) were used. Proteins were extracted using the IP lysis buffer or, for the extraction of brain proteins, the N-PER neuronal protein extraction reagent (Thermo Scientific, Waltham, MA, USA), containing protease inhibitors (Complete protease inhibitor cocktail, Roche). For pre-clearing, protein lysates (0.8–1 mg/mL total protein) were added to an Eppendorf tube containing Agarose beads (Protein A/G-Agarose (Santa Cruz, Carlsbad, CA, USA) (50 μL) and the mixture was incubated under rotation for 1–2 h at 4 °C and then centrifuged for 2 min at 2000× *g*. The resultant pre-cleared supernatant was incubated overnight with appropriate antibodies (mab2166, anti-polyglutamine 1C2, anti-STREP II-tag, anti-EGFP (both Abcam, Cambridge, UK)) at 4 °C under rotation. Next, 30 μL of Agarose beads were added for another 3 h after which the incubated lysate was centrifuged at 2000× *g* for 2 min at 4 °C and the supernatant was discarded. The pellet was washed 3 times in 1.0 mL ice-cold lysis-buffer. Finally, 25 μL of LDS-sample loading buffer (Invitrogen, Carlsbad, CA, USA) containing 50 mM Dithioerythritol (DTE) were added to the washed pellet. After boiling the sample at 90 °C for 5 min, the antigen was separated from the Agarose beads by centrifugation at 2000× *g* for 1 min at RT. TG6 was identified using a polyclonal TG6-specific antibody (1:500; Zedira, Darmstadt, Germany) for brain-derived TG6 or an anti-STREP II-tag antibody.

### 4.8. SDS-Gel Electrophoresis and Western Blot

Samples were loaded onto 3–8% linear gradient NuPage Trisacetate-polyacrylamide gels (Invitrogen, Carlsbad, CA, USA). Proteins were separated by electrophoresis (120V, constantly applied) and subsequently transferred onto a polyvinylidene fluoride (PVDF) membrane (MerckMillipore, Darmstadt, Germany) by the application of 15 V for 30 min. For immunodetection of (m)HTT and TG6, membranes were incubated overnight at 4 °C with antibody 2B7 (1:2000; CHDI Foundation) or an anti-TG6 antibody (polyclonal, 1:500, Zedira; anti-STREP-tag II, Abcam, Cambridge, UK), respectively. After 3 washing steps with TBST for 10 min each, the membrane was incubated with appropriate secondary goat anti-rabbit or goat anti-mouse antibodies (1:3000, Sigma Aldrich, St. Louis, MO, USA) coupled to alkaline-phosphatase- (AP) or horse-radish-peroxidase (HRP). Finally, the reactive bands were visualized using Sigma FAST BCIP/NBT tablets (Sigma Aldrich, St. Louis, MO, USA) or the Luminol chemiluminescence HRP-detection substrate (MerckMillipore, Darmstadt, Germany).

## 5. Conclusions

In summary, our data strongly suggest a prominent role for TG6 in the posttranslational modification of (m)HTT thus pointing away from TG2. Our results suggest that TG6 activity on mHTT may be causative for the modification of mHTT fragments that would be more prone to aggregation. Furthermore, the aggregation process seems to depend more on the regional distribution and physical proximity of transglutaminases and mHTT, rather than on differences in total protein amounts and enzymatic activity. Further studies analyzing the impact of TG6 knock-out/inactivation on mHTT aggregation and disease progression will finally elucidate the pathological relevance of our findings.

## Data Availability

The data presented in this study are available within the manuscript text, figures, and Supplementary Material.

## Supplementary Materials

The following are available online at https://www.mdpi.com/article/10.3390/ijms22168914/s1.
