# Supplementary material for: Transglutaminase 6 Is Colocalized and Interacts with Mutant Huntingtin in Huntington Disease Rodent Animal Models
Source: Int J Mol Sci. 2021 Aug 18;22(16):8914. doi: 10.3390/ijms22168914 (PMC8396294; doi:10.3390/ijms22168914)

International Journal of *Molecular Sciences*

MDPI

Supplementary Materials

Transglutaminase 6 Is Colocalized and Interacts with Mutant Huntingtin in Huntington Disease Rodent Animal Models

Anja Schulze-Krebs [1,*], Fabio Canneva [1], Judith Stemick [2], Anne-Christine Plank [1], Julia Harrer [1], Gillian P. Bates [3], Daniel Aeschlimann [4], Joan S. Steffan [5,6] and Stephan von Hörsten [1]

Table S1. Primary antibodies.

Antibody	Source	Epitope	mHTT Position
1C2	monoclonal	polyQ	polyglutamine stretch
2B7	monoclonal	MATLEKLMKAFESLKSF	1–17
S830	polyclonal	Exon1 (53Q)	1–90
mEM48	monoclonal	GST fusion protein from the first 256 amino acids from human huntingtin with the deletion of the polyglutamine tract.	1–256
MAB2166	monoclonal	GKVLLGEEEALEDDS	443–457
EP867Y	polyclonal	Synthetic peptide of aa 550–650	550–650
TG6	polyclonal	Human full-length neuronal transglutaminase recombinantly product in insect cells.	-
TG3	polyclonal	Human full-length epidermal transglutaminase recombinantly product in insect cells.	-
81D4	monoclonal	Synthetic peptide corresponding to N-epsilon-gamma glutamyl lysine (GGEL) bonds	-
STREP II Tag	monoclonal	ASWSHPQFEKGA	-
GFAP	polyclonal	Glial fibrillary acidic protein	-
NeuN	monoclonal	Neuron-Specific Nuclear Protein	-
GFP	polyclonal	Fusion protein corresponding to Aequorea Victoria GFP aa 1–246	-

Figure S1. TG-isoforms are widely distributed in the brains of rats.

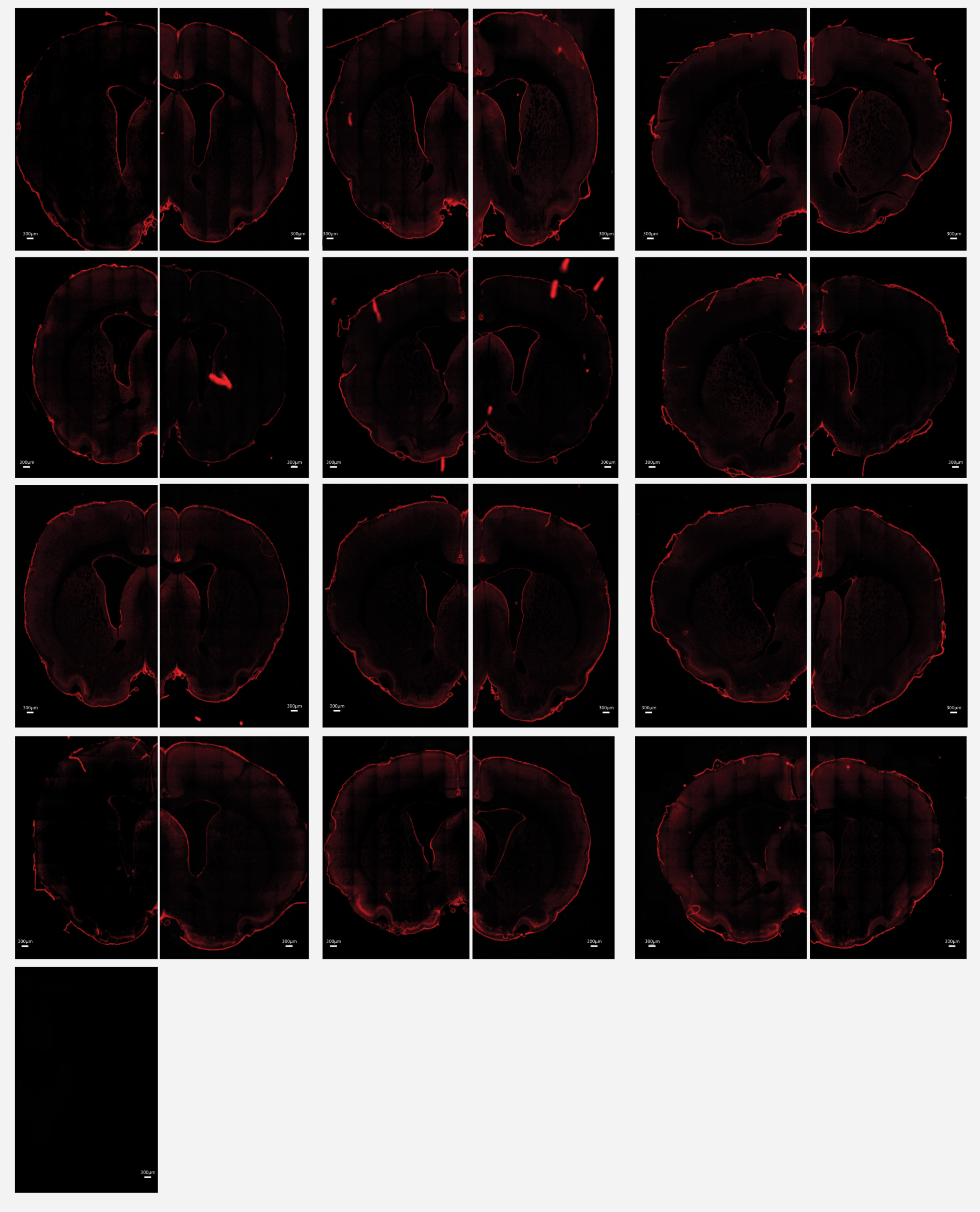

Figure S2. (m)HTT-immunopositive cells display a defined regional distribution pattern.

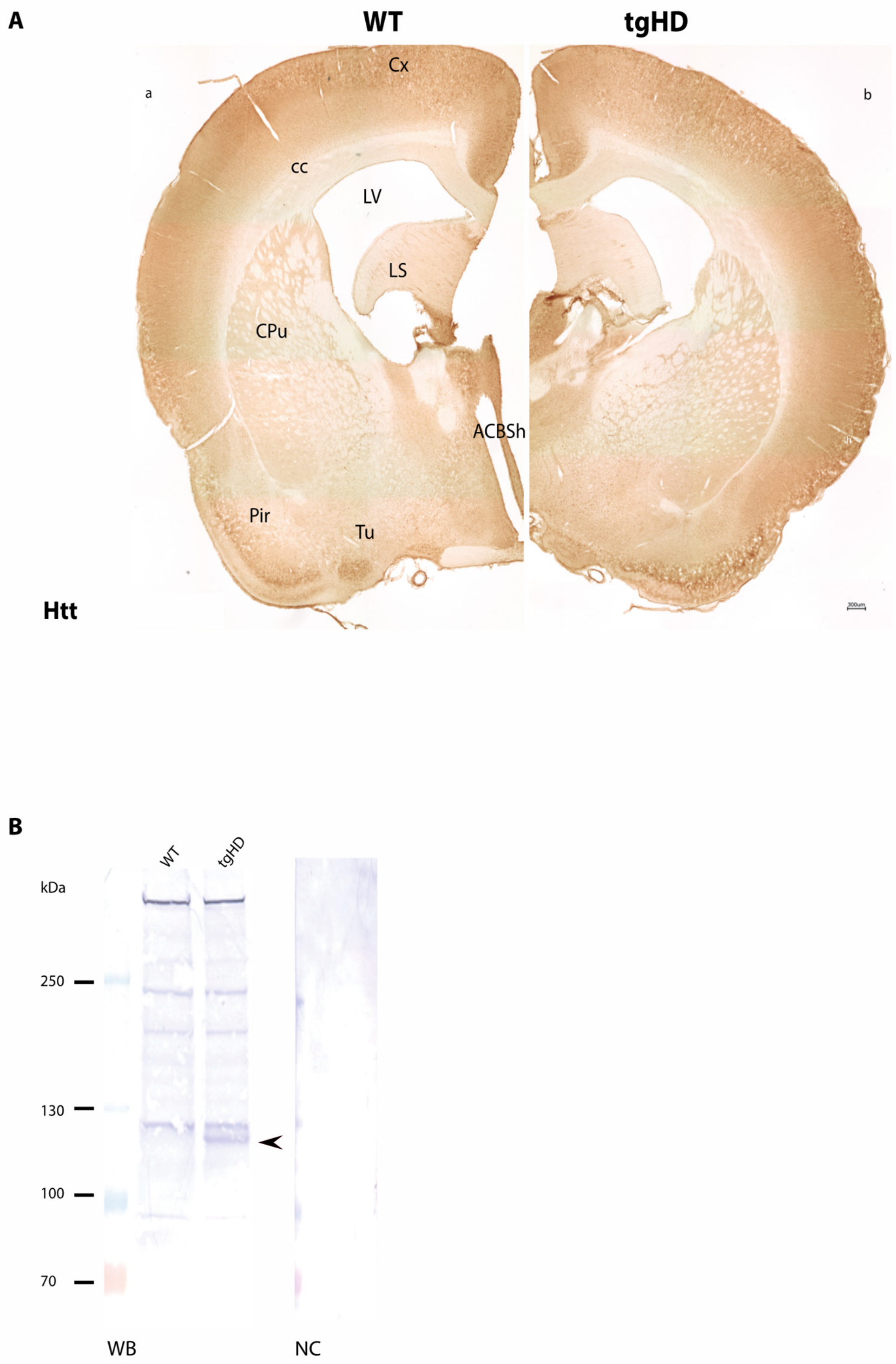

Figure S3. Transient transfection of SH-SY5Y cells with HTT exon 1 variants.

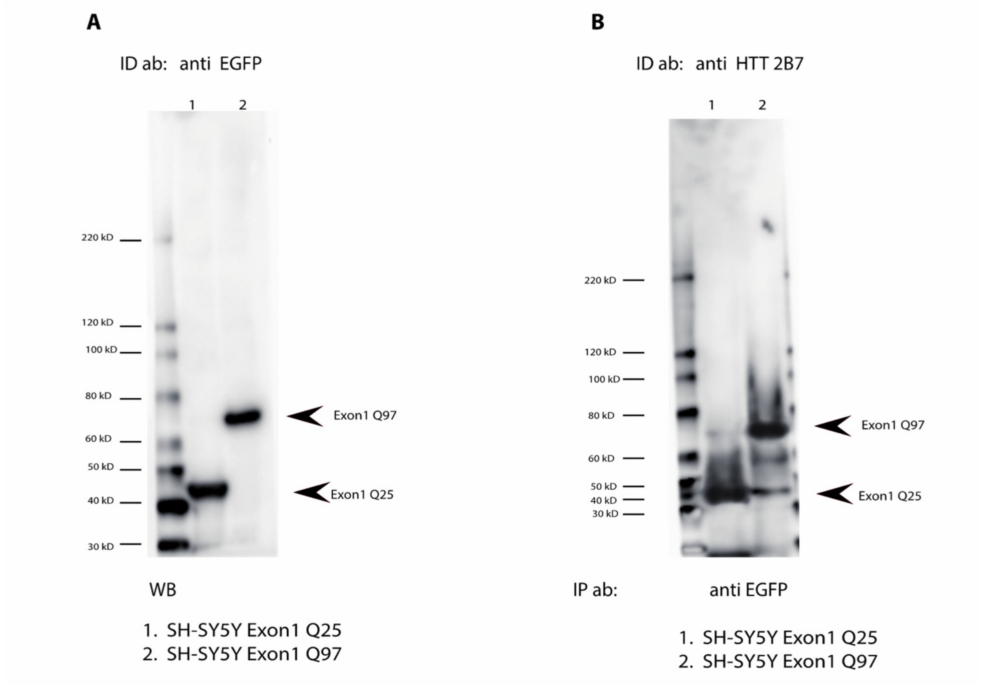

Figure S4. Representative images of double transfected (TG6, (m)HTT exon 1) aggregate-bearing SH-SY5Y cells.

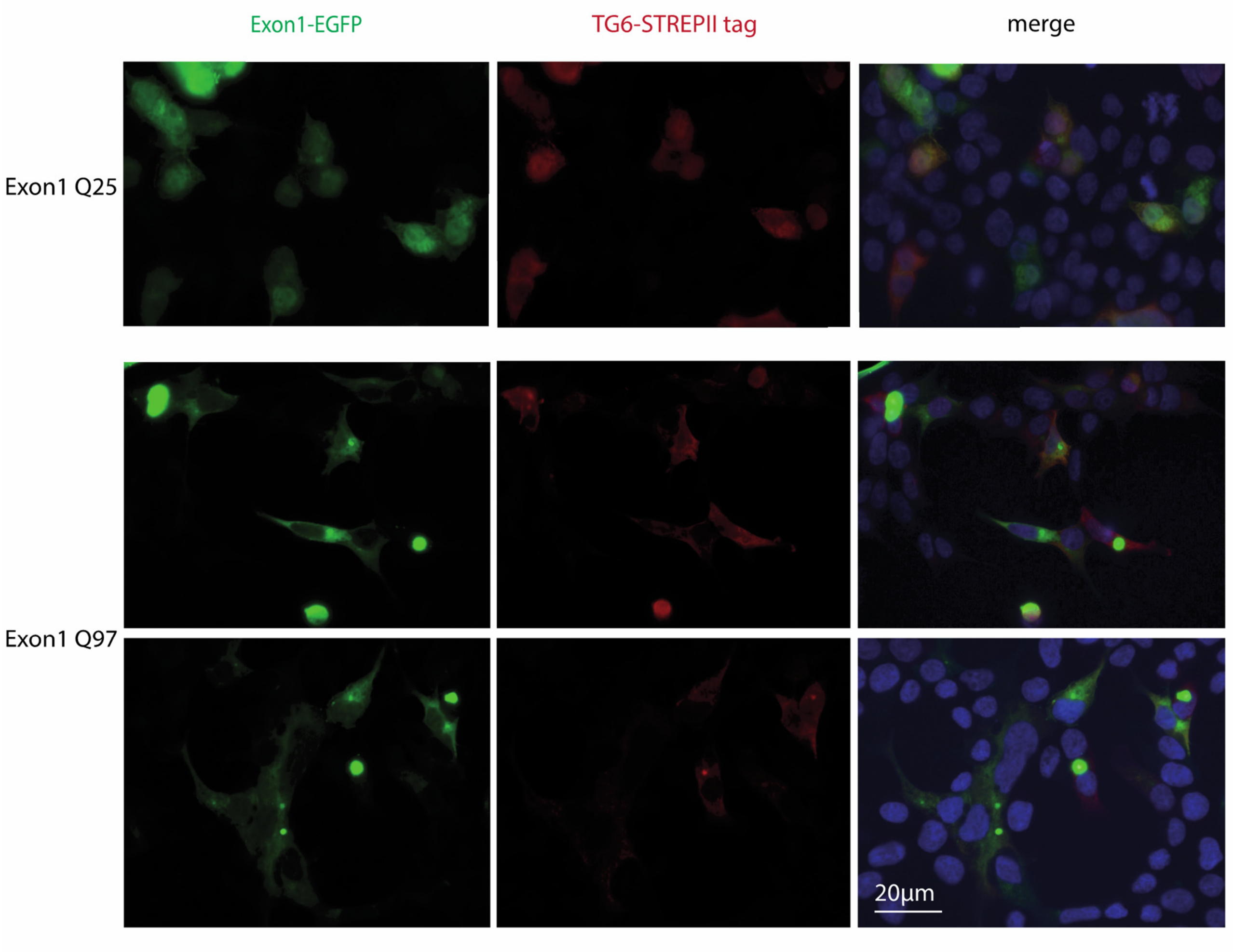

Supplement: Supplementary file 1 [file ijms-22-08914-s001.zip › ijms-1301688-supplementary.pdf]
